# Hypogammaglobulinemia in BLT Humanized Mice – An Animal Model of Primary Antibody Deficiency

**DOI:** 10.1371/journal.pone.0108663

**Published:** 2014-10-01

**Authors:** Francisco Martinez-Torres, Tomonori Nochi, Angela Wahl, J. Victor Garcia, Paul W. Denton

**Affiliations:** Division of Infectious Diseases, Department of Medicine, UNC Center for AIDS Research, University of North Carolina at Chapel Hill School of Medicine, Chapel Hill, North Carolina, United States of America; New York University, United States of America

## Abstract

Primary antibody deficiencies present clinically as reduced or absent plasma antibodies without another identified disorder that could explain the low immunoglobulin levels. Bone marrow-liver-thymus (BLT) humanized mice also exhibit primary antibody deficiency or hypogammaglobulinemia. Comprehensive characterization of B cell development and differentiation in BLT mice revealed other key parallels with primary immunodeficiency patients. We found that B cell ontogeny was normal in the bone marrow of BLT mice but observed an absence of switched memory B cells in the periphery. PC-KLH immunizations led to the presence of switched memory B cells in immunized BLT mice although plasma cells producing PC- or KLH- specific IgG were not detected in tissues. Overall, we have identified the following parallels between the humoral immune systems of primary antibody deficiency patients and those in BLT mice that make this *in vivo* model a robust and translational experimental platform for gaining a greater understanding of this heterogeneous array of humoral immunodeficiency disorders in humans: (i) hypogammaglobulinemia; (ii) normal B cell ontogeny in bone marrow; and (iii) poor antigen-specific IgG response to immunization. Furthermore, the development of strategies to overcome these humoral immune aberrations in BLT mice may in turn provide insights into the pathogenesis of some primary antibody deficiency patients which could lead to novel clinical interventions for improved humoral immune function.

## Introduction

Primary antibody deficiencies are characterized by reduced or absent plasma antibody levels when no other disorder can be identified as causing the immunoglobulin deficits [1], [2]. There are three major forms of primary antibody deficiency including X-linked or Bruton's agammaglobulinemia and selective IgA deficiency [1], [2]. The third and most frequently diagnosed form of this disease is common variable immunodeficiency (CVID) [3]–[5]. Both females and males are diagnosed with CVID, often during the second or third decade of life [6], [7]. Primary antibody deficiency in CVID results from aberrant B cell differentiation that could be due to many various genetic defects which may affect T, B and potentially other cells [3]–[5], [8]–[11]. Hypogammaglobulinemia in CIVD patients is manifested as profound reduction in serum IgG and low serum IgA, frequently accompanied by reduced serum IgM [12]. Clinical symptoms in these individuals include lymphoproliferation, recurrent respiratory bacterial infections (e.g., sinusitis, otitis media, bronchitis, and pneumonia), chronic diarrhea, granulomatous disease, autoimmune phenomena (e.g. immune thrombocytopenic purpura), malignancy and/or hepatitis [7], [13], [14]. Current standard of care for primary antibody deficiency patients is passive immunoglobulin transfer and treatment of recurrent infections with antimicrobials [15].

Due to the heterogeneous etiologies of primary antibody deficiency, these disorders are challenging to study in patients and to model *in vivo*. Patient-based research is primarily focused on peripheral blood analyses and is inherently accompanied by limitations that impede achieving adequately powered patient groups with specific diagnoses, especially given the factors which may preclude sufficient patient recruitment from within pediatric and adolescent populations [6], [7], [16], [17]. Moreover, effective translation of data from current mouse primary antibody deficiency models to human conditions has been limited by well described differences in the development and function of mouse B cells versus human B cells (reviewed in [18]–[20]). Nevertheless, a robust *in vivo* model of human primary antibody deficiency would serve two major purposes. First, the model would bolster efforts to understand the mechanisms responsible for the B cell compartment abnormalities in patients. Second, strategies to improve antibody production in primary antibody deficiency in patients could be evaluated in such a model for pre-clinical efficacy.

Humanized mice have the potential to serve as such a model. Humanized mice harbor *de novo* generated human immune cells that form a functional human immune system within each animal. Bone marrow-liver-thymus (BLT) humanized mice are generated by implantation of human thymus and liver tissues beneath the kidney capsule of immunodeficient mice followed by transplantation with autologous human CD34^+^ hematopoietic stem cells [21], [22]. BLT mice harbor robust levels of human hematopoietic cells (e.g. T cells, B cells, monocytes/macrophages and dendritic cells) throughout their body [22]–[26]. BLT mice have been extensively used for modeling HIV disease and other human specific conditions (reviewed in [27]). Nevertheless, humanized mice in general have exhibited relatively poor B cell function and rudimentary secondary lymphoid structure formation [21], [22], [25], [28]–[40]. Given the deficient humoral immune responses in humanized mice, despite their otherwise remarkable recapitulation of the human immune system's development and function, we examined the potential for BLT mice to serve as a primary antibody deficiency model. To do this we compared the human B cell development, differentiation and function we observed in BLT mice to literature detailing primary antibody deficiency in patients.

## Methods

### Ethics

Mice were maintained under specific pathogen-free conditions in accordance with protocols approved by the University of Texas Southwestern Medical Center at Dallas Institutional Animal Care and Use Committee in the UT Southwestern Animal Resources Center or in accordance with protocols approved by the University of North Carolina at Chapel Hill Institutional Animal Care and Use Committee in the UNC-CH Division of Laboratory Animal Medicine.

### Generation of BLT humanized mice, plasma immunoglobulin ELISA, PC-KLH immunization and plasma cell ELISPOT analyses

NOD/SCID IL-2Rγ^-/-^ (NOD.Cg-*Prkdc^scid^ Il2rγ^tm1Wjl^*/Szj; NSG) and BALB/c mice were obtained from The Jackson Laboratory. BLT mice were bioengineered with de-identified human tissues essentially as previously described [22], [24], [41]. Briefly, human fetal liver CD34^+^ cells were transplanted into preconditioned NSG mice implanted with autologous liver and thymus (Advanced Bioscience Resources). Peripheral blood plasma levels of human IgM, IgG and IgA in naïve BLT mice were measured by ELISA. In brief, 96-well ELISA plates (Thermo Scientific, Rochester NY) were coated with anti-human IgM, IgG or IgA antibodies (Bethyl Laboratories, Montgomery, TX) overnight at 4°C. After blocking with 1% BSA for 1 hour at room temperature (RT), the plates were treated with diluted plasma for 2 hours at RT and HRP-conjugated anti-human IgM, IgG or IgA antibodies (SouthernBiotech, Birmingham, AL) were then treated for 1 hour at RT. The reaction was developed with a TMB Microwell Peroxidase Substrate System (XPL, Gaithersburg, MD). Purified human IgM, IgG and IgA (Sigma, St Louis, MO) were used as standards.

BLT mice were immunized intraperitoneally with 100 µg of PC-KLH (Biosearch Technologies, Petaluma, CA) every other week for a total of four immunizations. Cholera toxin (1 µg) was used as an adjuvant (List Biological Laboratories, Campbell, CA). Antigen specific IgM and IgG were measured by ELISA and ELISPOT analyses. For ELISA analysis, 96-well ELISA plates were first pre-coated with PC-BSA (Biosearch Technologies, Petaluma, CA) or KLH (Sigma) overnight at 4°C, then the wells were blocked with 1% BSA. Following a 2 hour incubation with BLT mouse plasma at RT, wells were washed and treated with HRP-conjugated anti-human IgM or IgG antibodies for 1 hour at RT. Signal was developed with a TMB Microwell Peroxidase Substrate System. For the ELISPOT analysis, one week after the fourth immunization, bone marrow, spleen, lymph nodes, liver and lung were harvested from immunized BLT mice. Mononuclear cells were isolated from these tissues as we previously described [22]–[25] and evaluated by ELISPOT analysis for their antigen-specific Ig production. ELISPOT plates (EMD Millipore, Billerica, MA) were first pre-coated with PC-BSA or KLH overnight at 4°C. Cells freshly isolated from tissues of immunized BLT mice were suspended in RPMI-1640 containing 10% FBS, 100 I.U. penicillin and 100 µg/ml streptomycin and then 1×10^5^ cells in a total of 100 µl media were seeded per well and cultured for 4 hours in a 5% CO_2_ incubator. After washing, the wells were treated with HRP-conjugated anti-human IgM or IgG antibodies (SouthernBiotech) overnight at 4°C and the signals were then developed using AEC substrate (Sigma). Antibody positive cells were enumerated with an AID ELISPOT Reader System ELR04 (Strassberg, Germany).

### Human B cell flow cytometry panels

Flow cytometry data were collected using a BD FACSCanto cytometer and analyzed using BD FACSDiva software (v. 6.1.3). Flow cytometry antibody panels for analysis of B cells in BLT mice were: Panel A – IgD FITC (IA6-2), CD27 PE (L128), CD38 PerCP (HIT2), CD19 PE-Cy7 (SJ25C1), CD5 APC (UCHT2), CD45 APC-Cy7 (2D1); Panel B – TdT or IgG1 control FITC (HTdT-6; Supertechs), CD22 or IgG1k control PE (H1B22; Biolegend), CD20 PerCP (2H7), CD19 PE-Cy7 (SJ25C1), CD10 APC (H110a), CD45 APC-Cy7 (2D1); Panel C – CyIgM or IgG1k control FITC (G20-127), Cyλ5 or IgG1k control PerCP (courtesy of Dr. H. Karasuyama; Tokyo Medical and Dental University), CD19 PE-Cy7 (SJ25C1), CD10 APC (H110a), CD45 APC-Cy7 (2D1). Antibodies were purchased from BD Biosciences unless otherwise noted.

### Histological analyses

Tissues for histology were harvested from BLT and BALB/c mice, fixed in 4% paraformaldehyde for 24 hours at 4°C, embedded in paraffin, cut into 5 µm sections and mounted onto poly-L-lysine coated glass slides. Following paraffin removal, sections were stained with Mayer's hematoxylin and eosin (H&E) for analysis. Images were taken with an upright Nikon Microphot SA microscope with a DXM 1200 color camera and the white balance and brightness adjusted in Adobe Photoshop CS4.

### Statistics

Plots and descriptive statistics were generated in Prism v5 or v6 (Graph Pad). Values in text are presented as: (mean percent± standard deviation).

## Results

### BLT mice exhibit profound hypogammaglobulinemia

**Figure 1:**
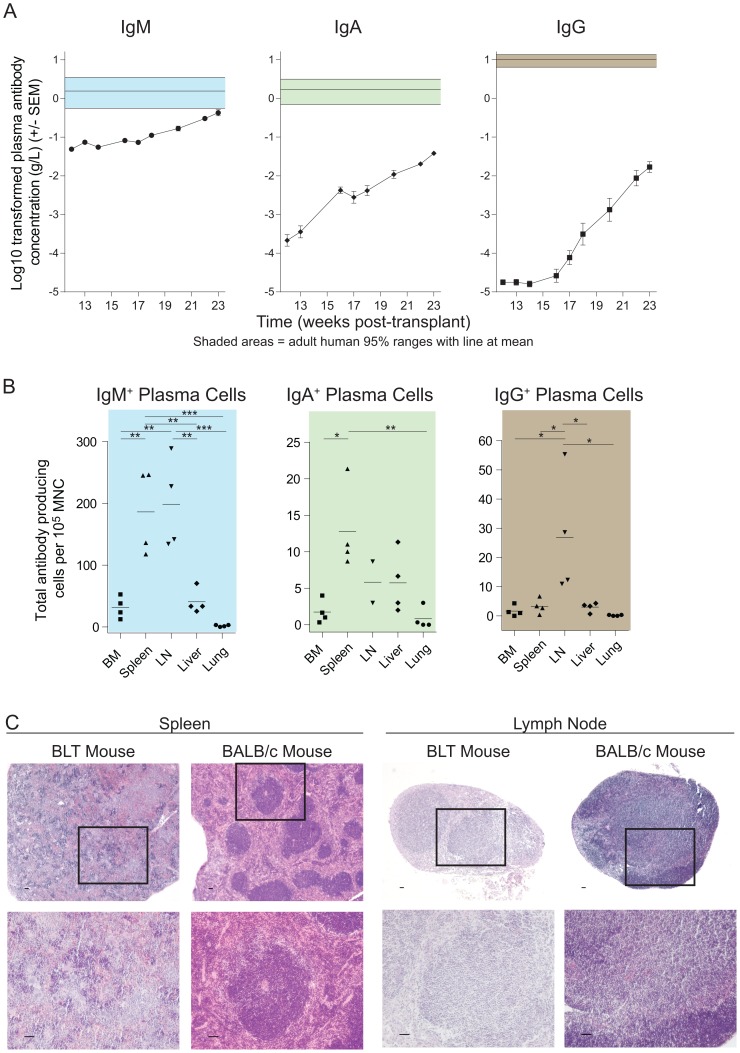
BLT humanized mice exhibit profound plasma hypogammaglobulinemia. (**A**) BLT mouse plasma (n = 13) was collected and analyzed by ELISA for the presence of human IgM, IgA and IgG. Log transformed plasma Ig levels in BLT mice are plotted on a linear y-axis and shown superimposed over the mean (center horizontal line) and 95% range (shaded area) Ig values from adult humans. (**B**) At harvest, ELISPOT analyses for plasma cells were performed with mononuclear cells isolated from the indicated tissues of BLT mice (n = 4). Statistical comparisons were 1 way ANOVA with Bonferroni's multiple comparisons tests. Plot colors are matched to the colors in (A). * indicates a p value less than 0.05. ** indicates a p value less than 0.01. *** indicates a p value less than 0.001. (**C**) Sections from the spleens (left) and lymph nodes (right) of BLT mice analyzed for lymphoid architecture via H&E staining. BALB/c tissues were included as positive controls for the appearance of normal immune structures. Scale bars  = 50 µm in all images. Boxes indicate the areas that are shown at higher magnification in the images below.

To bioengineer BLT humanized mice, human fetal liver CD34^+^ cells were transplanted into preconditioned NSG mice implanted with autologous liver and thymus. In total, 55 BLT mice generated using 20 different human donors were utilized herein. Peripheral blood human chimerism was assessed by flow cytometry in each BLT humanized mouse prior to use (week 16±4 weeks post-BLT humanization) [22], [23], [25], [26], [41]–[49]. We observed that 58% (±21%) of cells expressed hCD45; 42% (±19%) of hCD45^+^ cells also expressed hCD19; 43% (±23%) of hCD45^+^ cells also expressed hCD3 and 81% (±7%) of hCD3^+^ cells also expressed hCD4. To establish baseline characteristics of human Ig production in this model, we performed a longitudinal characterization of peripheral blood (PB) plasma levels of human IgM, IgA and IgG in BLT mice (n = 13) in the absence of a specific antigenic stimulus (referred to as “naïve” BLT mice). These values were then compared to 95% ranges for adult human levels for these immunoglobulins: IgM  = 0.56–3.52 g/L; IgA  = 0.70–3.12 g/L; and IgG  = 6.39–13.49 g/L [50]. Human IgM, human IgA and human IgG were all detected in BLT mice; however, their titers all remained below the human 95% range throughout the observation period (Figure 1A). To gain insights into the tissue source(s) of the antibodies present in BLT mouse plasma, we used ELISPOT analyses to quantitate the numbers of IgM, IgA and IgG producing plasma cells generating human antibodies in bone marrow (BM), spleen, lymph nodes (LN), liver and lungs. The results showed that there were more IgM-producing cells than IgA-producing and IgG-producing cells in BM, spleen, LN and liver (Figure 1B). Furthermore, we observed a lack of white pulp with germinal centers in the spleen and a lack of clear distinctions between the light and mantle zones in LN (Figure 1C) [51]. Despite this atypical tissue architecture in the spleen and LN, which could hinder isotype switching in B cells, these secondary lymphoid organs tissues were found to harbor the highest numbers of IgM, IgA and IgG plasma cells (Figure 1B).

### Classical Memory B cells were rare in the peripheral blood of BLT mice

**Figure 2:**
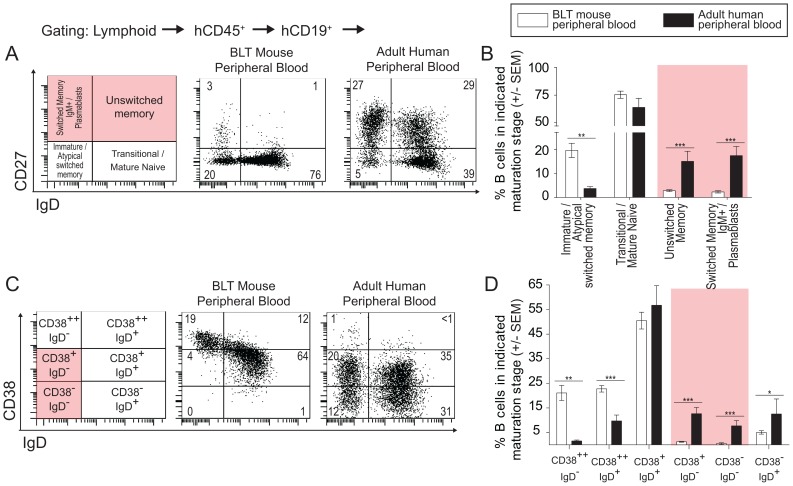
BLT mouse peripheral blood harbors few unswitched and switched memory B cells relative to adult human peripheral blood. (**A–B**) Representative flow cytometry analyses for CD27 and IgD expression on B cells in adult human PB and BLT mouse PB shows a paucity of unswitched/switched memory B cells in BLT mouse blood. (adult PB, n = 5; BLT PB, n = 18). (**C–D**) Flow cytometry plots and bar graph for CD38 and IgD expression on CD19^+^ cells reveal an absence of switched memory B cell populations in BLT mouse PB while these populations are present in adult human PB (adult PB, n = 5; BLT PB n = 16). * indicates a p value less than 0.05. ** indicates a p value less than 0.01. *** indicates a p value less than 0.001. Comprehensive statistical analyses are presented in Table 1.

We used complementary immunophenotyping strategies for assessing B cell differentiation. These strategies included examining combined surface expression patterns of: CD27 and IgD as markers of memory and maturation [52]–[54] and CD38 and IgD as classical clinical markers for distinguishing memory B cell subpopulations [53]. Using CD27 and IgD co-expression patterns, B cells were defined as immature/atypical switched memory B cells (CD27^neg^IgD^neg^), transitional and mature naïve (CD27^neg^IgD^+^), classical unswitched memory (CD27^+^IgD^+^), and classical switched memory IgM and plasmablasts (CD27^+^IgD^neg^) [52], [53]. The proportion of immature/atypical switched memory B cells in the PB of BLT mice (20%±12) was significantly greater than in normal adult human PB (4%±2) (p<0.05). Also, there were fewer unswitched memory (3%±2) and switched memory (2%±2) B cells in PB of BLT mice versus normal adult human PB which exhibited significantly more unswitched (15%±9) (p<0.001) and switched memory B cells (17%±9) (p<0.001) (Figure 2A–B; Table 1). Furthermore, the proportion of memory B cells in BLT mouse PB, recognized as such by their surface phenotypes [CD38^+^IgD^neg^ (1%±0.7) and CD38^neg^IgD^neg^ (0.5%±1)], were significantly reduced when compared to adult human PB (13%±6) (p<0.001) and (8%±5) (p<0.001), respectively (Figure 2C–D; Table 1). Integrated analysis of all three markers on BLT mouse peripheral blood B cells showed that <20% CD38^++^ IgD^neg^ cells were CD27^pos^ atypical switched memory cells while the majority were CD27^neg^ immature B cells [54], [55]. Overall, when compared to normal adult human PB, the reduced frequency of mature B cells and the paucity of classical unswitched/switched memory B cells in the PB of BLT mice indicate that the majority of B cells in these animals do not complete normal development.

**Table 1:** Comparison of B cell populations between BLT mouse and adult human peripheral blood using CD27/IgD and CD38/IgD co-expression patterns.

		BLT mouse PB	Adult human PB	*t*-test
CD27/IgD	Immature B/Atypical switched memory B	19.6±11.9 (n = 16)	3.7±1.9 (n = 5)	<0.01
	Transitional B/Mature Naïve B	75.4±13.0 (n = 16)	63.9±18.3 (n = 5)	n.s.
	Unswitched Memory B	2.8±1.5 (n = 16)	15.0±9.4 (n = 5)	<0.001
	Switched Memory IgM+ B/Plasmablasts	2.3±1.9 (n = 16)	17.4±8.6 (n = 5)	<0.001
CD38/IgD	CD38^neg^ IgD^+^	5.0±2.8 (n = 16)	12.5±13.6 (n = 5)	<0.05
	CD38^+^ IgD^+^	50.5±13.6 (n = 16)	56.8±17.5 (n = 5)	n.s.
	CD38^++^ IgD^+^	22.8±5.3 (n = 16)	10.0±5.3 (n = 5)	<0.001
	CD38^++^ IgD^neg^	21.1±12.2 (n = 16)	1.6±0.9 (n = 5)	<0.01
	CD38^+^ IgD^neg^	1.3±0.7 (n = 16)	12.6±5.7 (n = 5)	<0.001
	CD38^neg^ IgD^neg^	0.5±1.3 (n = 16)	7.7±4.9 (n = 5)	<0.001

Data presented as Mean ± SD.

n.s.  =  not significantly different.

Immature B/Atypical switched memory B =  CD27^neg^ IgD^neg^.

Naïve Mature B =  CD27^neg^ IgD^+^.

Transitional B/Mature Naïve B =  CD27^+^ IgD^+^.

Switched Memory IgM+ B/Plasmablasts  =  CD27^+^ IgD^neg^.

### B cell ontogeny in BM progresses normally in BLT mice, but B cells do not mature into memory cells following BM emigration

**Figure 3:**
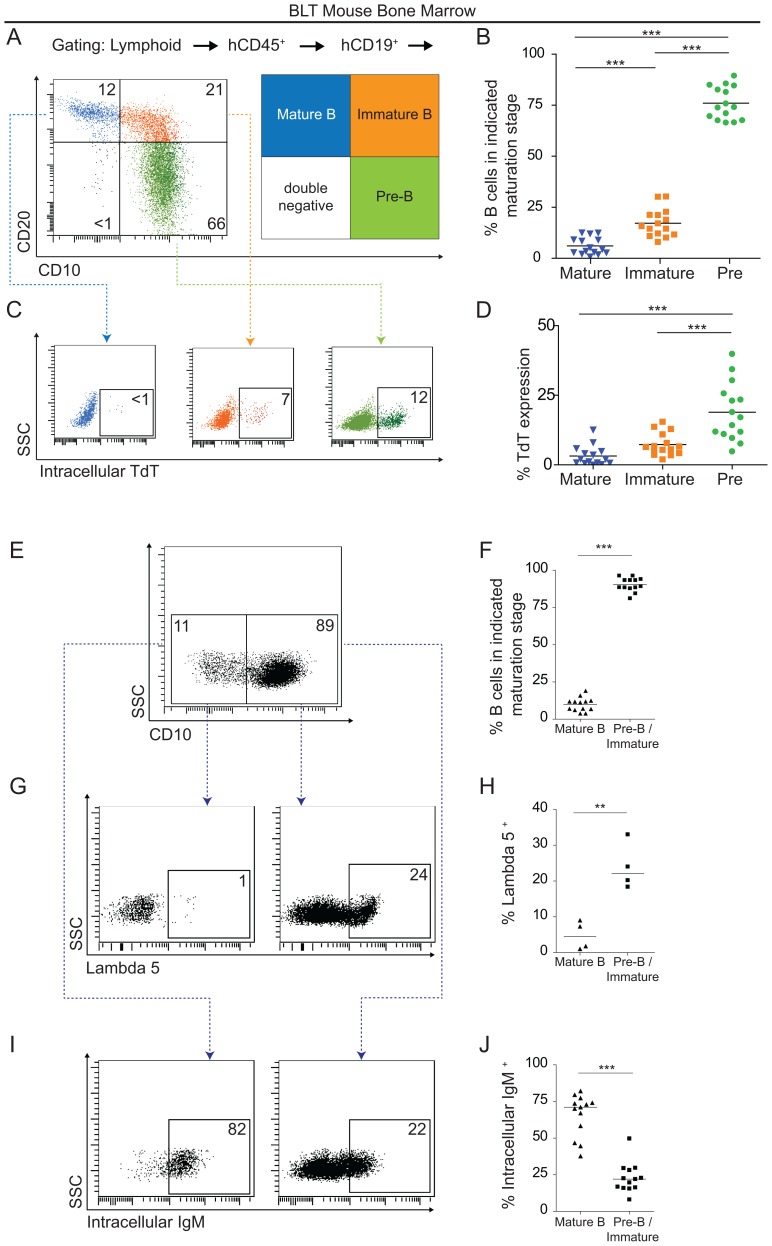
B cell ontogeny in the bone marrow of BLT mice follows canonical patterns. (**A–B**) Flow cytometry and scatter plots reveal the relative paucity of mature B cells in the BM as defined according to CD10 and CD20 expression. (n = 15; 1 way ANOVA with Bonferroni's multiple comparisons tests). (**C–D**) Flow cytometry and scatter plots reveal that, as expected, immature B cells exhibited higher levels intracellular TdT expression, a protein that is active during B cell receptor rearrangement. (n = 15; 1 way ANOVA with Bonferroni's multiple comparisons tests). (**E–F**) Flow cytometry and scatter plots for CD10 expression show that the majority of BLT mouse BM CD19^+^ cells are Pre-B/Immature B cells. (n = 13, *t*-test). (**G–H**) More BLT mouse Pre-B/Immature B cells identified in (E–F) express the pre-B cell receptor surrogate light chain component lambda 5 than CD10^neg^ mature B cells. (n = 4, *t*-test). (**I–J**) Conversely, more CD10^neg^ mature B cells expressed intracellular IgM than did the Pre-B/Immature B cells present in BLT mouse BM (n = 13, *t*-test). ** indicates a p value less than 0.01. *** indicates a p value less than 0.001.

Since the BM is the primary lymphoid tissue where B cell development occurs, we analyzed B cell ontogeny in BLT mouse BM using surface and intracellular marker expression [56], [57]. We found that CD10^+^CD20^neg^ pre-B cells, were the predominant population in the BM of BLT mice (76%±8) followed by CD10^+^CD20^+^ immature B cells (17%±7) (p<0.001) and CD10^neg^CD20^+^ mature B cells (6%±4) (p<0.001) (Figure 3A–B). The intracellular expression of terminal deoxynucleotide transferase (TdT) is a phenotypic marker for early stages of B cell development [58]. Accordingly, the intracellular expression of TdT was greater in BLT mouse BM CD10^+^CD20^neg^ pre-B cells (19%±10) when compared to CD10^+^CD20^+^ immature-B cells (7%±4) (p<0.001) or CD10^neg^ CD20^+^ mature B cells (3%±3) (p<0.001) (Figure 3C–D). We also analyzed the expression of the pre-B cell receptor surrogate light chain component λ5 [59] and the intracellular expression of IgM [56] in BM B cells. The majority of B cells were CD10^+^ pre-B cells/immature B cells (90%±5) while the remaining B cells were mature CD10^neg^ B cells (10%±5) (p<0.001) (Figure 3E–F). A greater proportion of pre-B cells/immature B cells expressed λ5 (24%±7) in comparison to mature B cells (5%±4) (p<0.01) (Figure 3G–H) and the inverse was observed regarding intracellular IgM expression. Specifically, the proportion of CD10^+^ pre-B cells/immature B cells expressing intracellular IgM was significantly lower (23%±10) compared to CD10^neg^ mature B cells (66%±14) (p<0.001) (Figure 3I–J). These data show that B cell ontogeny in the BM of BLT mice progresses as described for human BM.

**Figure 4:**
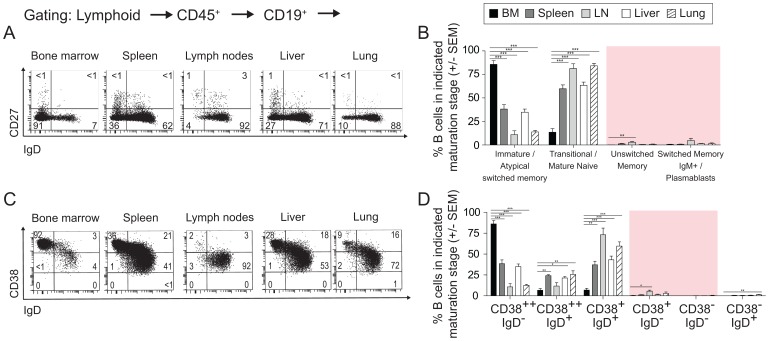
Most B cells in BLT mouse tissues exhibit an immature phenotype. (**A–B**) Flow cytometry plots and bar graph for CD27 and IgD expression on B cells reveal an absence of unswitched/switched memory B cells in BLT mouse BM, spleen, LN, liver and lungs (n = 5 for all tissues). (**C–D**) Flow cytometry plots and bar graph for CD38 and IgD expression on CD19^+^ cells reveal an absence of switched memory B cell populations in BLT mouse tissues (n = 5 for all tissues). * indicates a p value less than 0.05. ** indicates a p value less than 0.01. *** indicates a p value less than 0.001. Comprehensive statistical analyses are presented in Table 2.

Despite normal ontogeny, B cell maturation and development in BLT mice appeared to progress to either the pre-naïve or mature naïve stage and then stall. CD27/IgD co-expression patterns revealed that both unswitched and switched memory B cells were rare in BLT mouse BM (0.1%±0.1; 0.6%±0.3), spleen (1%±1; 0.7%±0.6), LN (3%±1.7; 5%±5), liver (0.5%±0.2; 1.3%±0.8) and lung (0.7%±0.7; 1%±2) (Figure 4A–B; Table 2). These systemic observations were confirmed with a complementary staining strategy. Specifically, memory B cells (defined as CD38^+^ IgD^neg^ and CD38^neg^ IgD^neg^) were rare in the BM, spleen, LN, liver and lung of BLT mice (<1% in all tissues) (Figure 4E–F; Table 2). These data, together with the PB data presented above, indicate that human B cells in BLT mice successfully emigrate from the BM to the periphery where they fail to mature into classical unswitched/switched memory B cells. This stall in B cell differentiation in BLT mice could result from limited antigenic stimulation associated with their specific pathogen-free housing conditions [22], [28].

**Table 2:** B cell populations in BLT mice according to CD27/IgD and CD38/IgD co-expression patterns.

		Tissue	BLT Mice	1 way ANOVA Bonferroni's multiple comparisons tests	
CD27/IgD	Immature B/Atypical switched memory B	BM	85.7±8.8 (n = 5)	BM vs. Spleen: <0.001	Spleen vs. Liver: n.s.
		Spleen	38.2±10.3 (n = 5)	BM vs. LN: <0.001	Spleen vs. Lung: <0.01
		LN	11.0±9.4 (n = 5)	BM vs. Liver: <0.001	LN vs. Liver: <0.01
		Liver	34.8±7.6 (n = 5)	BM vs. Lung: <0.001	LN vs. Lung: n.s.
		Lung	13.9±3.5 (n = 5)	Spleen vs. LN: <0.001	Liver vs. Lung: <0.01
	Transitional B/Mature Naïve B	BM	13.5±8.7 (n = 5)	BM vs. Spleen: <0.001	Spleen vs. Liver: n.s.
		Spleen	59.8±9.2 (n = 5)	BM vs. LN: <0.001	Spleen vs. Lung: <0.01
		LN	81.3±11.2 (n = 5)	BM vs. Liver: <0.001	LN vs. Liver: <0.05
		Liver	63.5±7.7 (n = 5)	BM vs. Lung: <0.001	LN vs. Lung: n.s.
		Lung	84.2±5.7 (n = 5)	Spleen vs. LN: <0.01	Liver vs. Lung: <0.05
	Unswitched Memory B	BM	0.1±0.1 (n = 5)	BM vs. Spleen: n.s.	Spleen vs. Liver: n.s.
		Spleen	1.2±1.0 (n = 5)	BM vs. LN: <0.01	Spleen vs. Lung: n.s.
		LN	3.0±1.7 (n = 5)	BM vs. Liver: n.s.	LN vs. Liver: <0.01
		Liver	0.5±0.2 (n = 5)	BM vs. Lung: n.s.	LN vs. Lung: <0.05
		Lung	0.7±0.7 (n = 5)	Spleen vs. LN: n.s.	Liver vs. Lung: n.s.
	Switched Memory IgM+ B/Plasmablasts	BM	0.6±0.3 (n = 5)	BM vs. Spleen: n.s.	Spleen vs. Liver: n.s.
		Spleen	3.7±0.6 (n = 5)	BM vs. LN: n.s.	Spleen vs. Lung: n.s.
		LN	4.7±4.9 (n = 5)	BM vs. Liver: n.s.	LN vs. Liver: n.s.
		Liver	1.3±0.8 (n = 5)	BM vs. Lung: n.s.	LN vs. Lung: n.s.
		Lung	1.3±2.0 (n = 5)	Spleen vs. LN: n.s.	Liver vs. Lung: n.s.
CD38/IgD	CD38^neg^ IgD^+^	BM	0.1±0.1 (n = 5)	BM vs. Spleen: n.s.	Spleen vs. Liver: n.s.
		Spleen	0.6±0.4 (n = 5)	BM vs. LN: n.s.	Spleen vs. Lung: n.s.
		LN	0.5±0.4 (n = 5)	BM vs. Liver: n.s.	LN vs. Liver: n.s.
		Liver	0.6±0.4 (n = 5)	BM vs. Lung: <0.01	LN vs. Lung: n.s.
		Lung	1.3±0.8 (n = 5)	Spleen vs. LN: n.s.	Liver vs. Lung: n.s.
	CD38^+^ IgD^+^	BM	6.9±4.5 (n = 5)	BM vs. Spleen: <0.01	Spleen vs. Liver: n.s.
		Spleen	37.1±9.5 (n = 5)	BM vs. LN: <0.001	Spleen vs. Lung: n.s.
		LN	73.2±17.7 (n = 5)	BM vs. Liver: <0.001	LN vs. Liver: <0.01
		Liver	43.2±10.0 (n = 5)	BM vs. Lung: <0.001	LN vs. Lung: n.s.
		Lung	59.6±11.1 (n = 5)	Spleen vs. LN: <0.001	Liver vs. Lung: n.s.
	CD38^++^ IgD^+^	BM	6.8±4.4 (n = 5)	BM vs. Spleen: <0.01	Spleen vs. Liver: n.s.
		Spleen	24.2±2.7 (n = 5)	BM vs. LN: n.s.	Spleen vs. Lung: n.s.
		LN	11.7±9.0 (n = 5)	BM vs. Liver: <0.05	LN vs. Liver: n.s.
		Liver	21.4±4.0 (n = 5)	BM vs. Lung: <0.01	LN vs. Lung: <0.05
		Lung	25.6±9.9 (n = 5)	Spleen vs. LN: n.s.	Liver vs. Lung: n.s.
	CD38^++^ IgD^neg^	BM	86.1±9.0 (n = 5)	BM vs. Spleen: <0.001	Spleen vs. Liver: n.s.
		Spleen	38.5±10.2 (n = 5)	BM vs. LN: <0.001	Spleen vs. Lung: <0.001
		LN	10.8±8.6 (n = 5)	BM vs. Liver: <0.001	LN vs. Liver: <0.01
		Liver	35.0±7.2 (n = 5)	BM vs. Lung: <0.001	LN vs. Lung: n.s.
		Lung	12.6±2.8 (n = 5)	Spleen vs. LN: <0.001	Liver vs. Lung: <0.01
	CD38^+^ IgD^neg^	BM	0.4±0.3 (n = 5)	BM vs. Spleen: n.s.	Spleen vs. Liver: n.s.
		Spleen	1.2±0.4 (n = 5)	BM vs. LN: <0.05	Spleen vs. Lung: n.s.
		LN	5.6±2.9 (n = 5)	BM vs. Liver: n.s.	LN vs. Liver: n.s.
		Liver	1.5±1.1 (n = 5)	BM vs. Lung: n.s.	LN vs. Lung: n.s.
		Lung	2.9±3.8 (n = 5)	Spleen vs. LN: <0.05	Liver vs. Lung: n.s.
	CD38^neg^ IgD^neg^	BM	0.0±0.0 (n = 5)	BM vs. Spleen: n.s.	Spleen vs. Liver: n.s.
		Spleen	0.0±0.0 (n = 5)	BM vs. LN: n.s.	Spleen vs. Lung: n.s.
		LN	0.1±0.2 (n = 5)	BM vs. Liver: n.s.	LN vs. Liver: n.s.
		Liver	0.1±0.2 (n = 5)	BM vs. Lung: n.s.	LN vs. Lung: n.s.
		Lung	0.4±0.7 (n = 5)	Spleen vs. LN: n.s.	Liver vs. Lung: n.s.

Data presented as Mean ± SD.

n.s.  =  not significantly different.

Immature B/Atypical switched memory B =  CD27^neg^ IgD^neg^.

Naïve Mature B =  CD27^neg^ IgD^+^.

Transitional B/Mature Naïve B =  CD27^+^ IgD^+^.

Switched Memory IgM + B/Plasmablasts  =  CD27^+^ IgD^neg^.

### Immunization led to increased numbers of switched memory B cells in BLT mouse tissues

**Figure 5:**
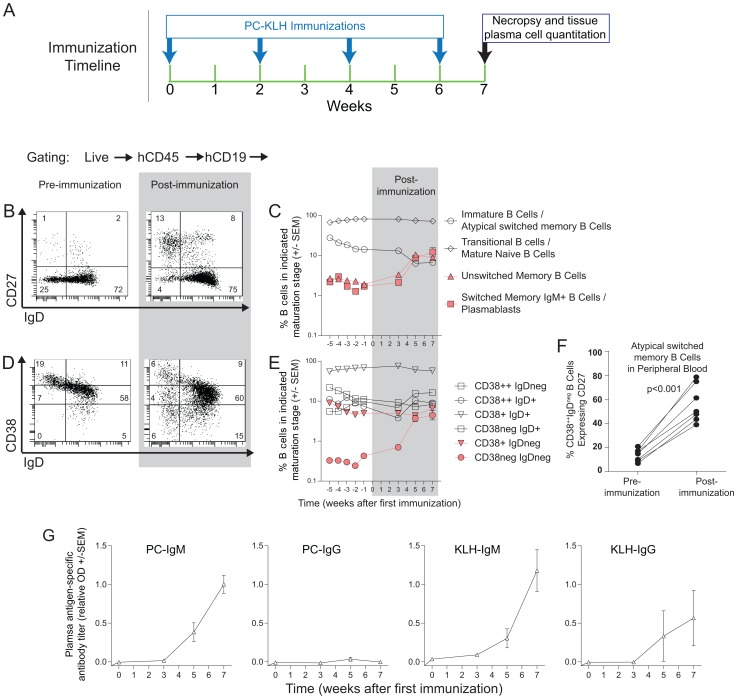
Repeated immunizations with PC-KLH induced B cell differentiation in BLT mouse peripheral blood towards mature phenotypes and increased plasma antigen-specific IgM. (**A**) Immunization schedule. (**B–E**) The B cell populations in the PB of BLT mice (n = 7) were characterized before, during and after repeated PC-KLH immunizations. Representative flow cytometry analyses for CD27 and IgD expression on PB B cells (B) and longitudinal analysis of B cell populations (C) are shown. Similarly, (D–E) depict representative flow cytometry and longitudinal data regarding CD38 and IgD expression of PB B cells. (**F**) The percentage of CD38^++^IgD^neg^ B cells that also express CD27 was calculated pre- and post-immunization to determine the contribution of atypical switched memory B cells to the CD27^neg^IgD^neg^ population in (B). (**G**) Plasma levels of antigen-specific IgM and IgG were measured longitudinally throughout the course of repeated PC-KLH immunizations (n = 7).

To test the hypothesis that antibody production in BLT mice could be improved with specific antigen stimulation, BLT mice were immunized intraperitoneally with conjugated phosphorylcholine - keyhole limpet hemocyanin (PC-KLH [60]) every other week for a total of four immunizations (Figure 5A). During the immunization series, we analyzed the peripheral B cell populations longitudinally and observed gradual increases in PB unswitched and classical switched memory B cell levels following immunization (Figure 5B–C). Furthermore, examinations of B cells before and after immunization according to CD38 and IgD co-expression patterns indicated that mature and memory B cell populations increased in the peripheral blood as a result of repeated immunizations (Figures 5D–E; Table 3). Integrated analysis of all three markers also showed an increased proportion of atypical switched memory cells (i.e. CD38^++^ IgD^neg^ cells expressing CD27) in the peripheral blood following the immunizations (Figures 5F) [54], [55]. The classical and atypical switched memory cells present in the peripheral blood following immunization likely account for the antigen-specific antibodies present in plasma following immunization (Figures 5G).

**Table 3:** Comparison of B cell populations between naïve and PC-KLH immunized BLT mice using CD27/IgD and CD38/IgD co-expression patterns.

		Tissue	BLT Mice*	Immunized BLT Mice	*t*-test
CD27/IgD	Immature B/Atypical switched memory B	BM	85.7±8.8 (n = 5)	82.4±13.4 (n = 7)	n.s.
		Spleen	38.2±10.3 (n = 5)	32.9±16.4 (n = 7)	n.s.
		LN	11.0±9.4 (n = 5)	11.0±7.6 (n = 7)	n.s.
		Liver	34.8±7.6 (n = 5)	38.7±16.4 (n = 7)	n.s.
		Lung	13.9±3.5 (n = 5)	17.3±6.6 (n = 7)	n.s.
	Transitional B/Mature Naïve B	BM	13.5±8.7 (n = 5)	11.7±12.7 (n = 7)	n.s.
		Spleen	59.8±9.2 (n = 5)	23.8±29.3 (n = 7)	<0.05
		LN	81.3±11.2 (n = 5)	70.1±11.6 (n = 7)	n.s.
		Liver	63.5±7.7 (n = 5)	39.1±23.1 (n = 7)	<0.05
		Lung	84.2±5.7 (n = 5)	56.0±20.9 (n = 7)	<0.05
	Unswitched Memory B	BM	0.1±0.1 (n = 5)	0.6±0.4 (n = 7)	<0.01
		Spleen	1.2±1.0 (n = 5)	5.6±5.2 (n = 7)	n.s.
		LN	3.0±1.7 (n = 5)	8.0±2.7 (n = 7)	<0.01
		Liver	0.5±0.2 (n = 5)	1.6±0.7 (n = 7)	<0.01
		Lung	0.7±0.7 (n = 5)	3.6±3.5 (n = 7)	n.s.
	Switched Memory IgM + B/Plasmablasts	BM	0.6±0.3 (n = 5)	5.3±3.2 (n = 7)	<0.01
		Spleen	3.7±0.6 (n = 5)	37.7±23.2 (n = 7)	<0.01
		LN	4.7±4.9 (n = 5)	10.9±6.5 (n = 7)	n.s.
		Liver	1.3±0.8 (n = 5)	20.7±14.1 (n = 7)	<0.05
		Lung	1.3±2.0 (n = 5)	23.2±17.0 (n = 7)	<0.05
CD38/IgD	CD38^neg^ IgD^+^	BM	0.1±0.1 (n = 5)	2.1±2.6 (n = 7)	n.s.
		Spleen	0.6±0.4 (n = 5)	3.6±4.6 (n = 7)	n.s.
		LN	0.5±0.4 (n = 5)	9.2±5.7 (n = 7)	<0.01
		Liver	0.6±0.4 (n = 5)	7.8±5.5 (n = 7)	<0.05
		Lung	1.3±0.8 (n = 5)	13.1±10.9 (n = 7)	<0.05
	CD38^+^ IgD^+^	BM	6.9±4.5 (n = 5)	8.4±8.6 (n = 7)	n.s.
		Spleen	37.1±9.5 (n = 5)	20.8±22.9 (n = 7)	n.s.
		LN	73.±17.7 (n = 5)	67.3±9.1 (n = 7)	n.s.
		Liver	43.2±10.0 (n = 5)	30.6±16.9 (n = 7)	n.s.
		Lung	59.6±11.1 (n = 5)	41.9±17.2 (n = 7)	n.s.
	CD38^++^ IgD^+^	BM	6.8±4.4 (n = 5)	2.3±2.3 (n = 7)	<0.05
		Spleen	24.2±2.7 (n = 5)	6.1±3.8 (n = 7)	<0.001
		LN	11.7±9.0 (n = 5)	8.1±5.1 (n = 7)	<0.05
		Liver	21.4±4.0 (n = 5)	50.3±23.5 (n = 7)	<0.001
		Lung	25.6±9.9 (n = 5)	25.7±22.9 (n = 7)	<0.01
	CD38^++^ IgD^neg^	BM	86.1±9.0 (n = 5)	86.0±13.9 (n = 7)	n.s.
		Spleen	38.5±10.2 (n = 5)	63.2±27.1 (n = 7)	n.s.
		LN	10.8±8.6 (n = 5)	8.1±5.1 (n = 7)	n.s.
		Liver	35.0±7.2 (n = 5)	50.3±23.5 (n = 7)	n.s.
		Lung	12.6±2.8 (n = 5)	25.7±22.9 (n = 7)	n.s.
	CD38^+^ IgD^neg^	BM	0.4±0.3 (n = 5)	1.4±1.0 (n = 7)	n.s.
		Spleen	1.2±0.4 (n = 5)	6.3±4.5 (n = 7)	<0.05
		LN	5.6±2.9 (n = 5)	11.8±7.9 (n = 7)	n.s.
		Liver	1.5±1.1 (n = 5)	7.1±4.2 (n = 7)	<0.05
		Lung	2.9±3.8 (n = 5)	9.3±7.0 (n = 7)	n.s.
	CD38^neg^ IgD^neg^	BM	0.0±0.0 (n = 5)	0.4±0.3 (n = 7)	<0.05
		Spleen	0.0±0.0 (n = 5)	1.4±0.8 (n = 7)	<0.01
		LN	0.1±0.2 (n = 5)	1.7±0.5 (n = 7)	<0.001
		Liver	0.1±0.2 (n = 5)	5.3±4.1 (n = 7)	<0.05
		Lung	0.4±0.7 (n = 5)	13.1±10.9 (n = 7)	<0.05

*Data for BLT mice also included in Table 2.

Data presented as Mean ± SD.

n.s.  =  not significantly different.

Immature B/Atypical switched memory B =  CD27^neg^ IgD^neg^.

Naïve Mature B =  CD27^neg^ IgD^+^.

Transitional B/Mature Naïve B =  CD27^+^ IgD^+^.

Switched Memory IgM + B/Plasmablasts  =  CD27^+^ IgD^neg^.

**Figure 6:**
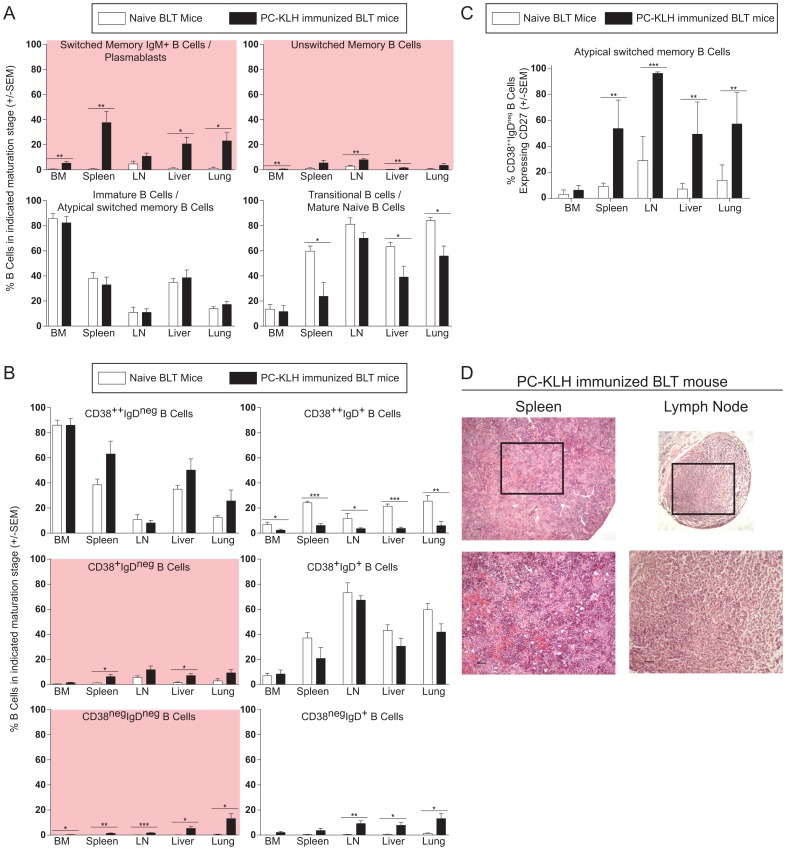
Repeated immunizations with PC-KLH induced B cell differentiation in BLT mouse tissues towards mature phenotypes. (**A–B**) B cell populations in the BM, spleen, LN, liver and lungs were characterized in naïve and immunized BLT mice. CD27 and IgD (A) (naïve, n = 5; immunized, n = 7 for all tissues; *t*-tests) and CD38 and IgD (C) (naïve, n = 5; immunized, n = 7; *t*-tests) co-expression patterns revealed increased levels of CD19^+^ cells exhibiting mature phenotypes throughout the body following the repeated PC-KLH immunizations. (**C**) The percentage of CD38^++^IgD^neg^ B cells that also express CD27 was calculated in naive BLT mice and PC-KLH immunized BLT mice to determine the contribution of atypical switched memory B cells to the CD27^neg^IgD^neg^ populations from each tissue in (A). (**D**) Sections from spleen (left) and lymph node (right) of PC-KLH immunized BLT mice analyzed for lymphoid architecture via H&E staining. Scale bars  =  50 µm in all images. Boxes indicate the areas that are shown at higher magnification in the images below. * indicates a p value less than 0.05. ** indicates a p value less than 0.01. *** indicates a p value less than 0.001. Comprehensive statistical analyses are presented in Table 3.

Next, we analyzed tissues of immunized BLT mice and compared their B cells to those derived from naive BLT mice. We observed that memory B cell numbers increased in each tissue evaluated versus non-immunized animals (Figure 6A; Table 3). Similarly, CD38 and IgD co-expression patterns indicated that mature and memory B cell populations increased throughout the body as a result of the immunizations (Figures 6B; Table 3). Moreover, integrated analysis of all three markers also showed an increased proportion of atypical switched memory cells (i.e. CD38^++^ IgD^neg^ cells expressing CD27) in the spleen, LN, liver and lung following the immunizations (Figures 6C) [54], [55]. However, we did not observe typical lymphoid architecture in the secondary lymphoid tissues of immunized NSG-BLT mice (Figures 6D).

**Figure 7:**
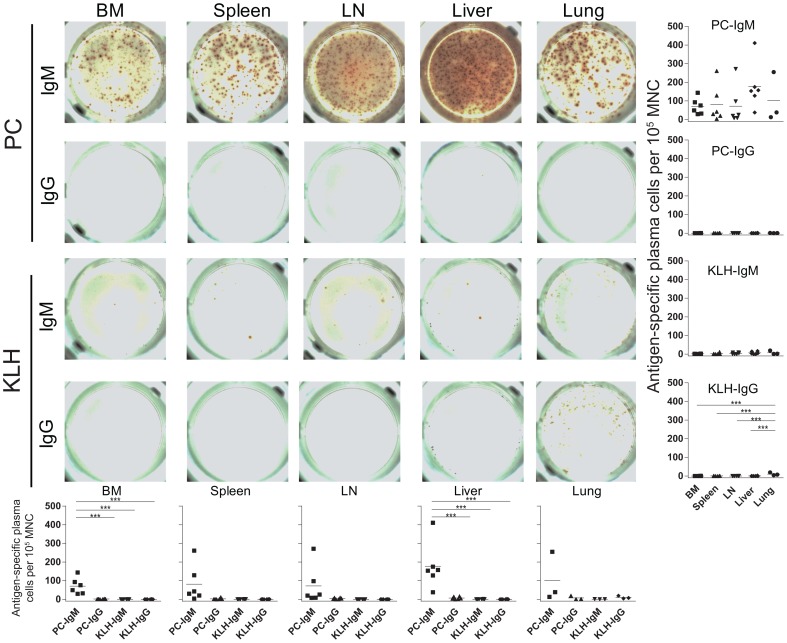
PC-KLH induced systemic production of PC-specific IgM expressing plasma cells in BLT mice. ELISPOT analyses revealed the presence of PC-specific IgM producing plasma cells in the BM (n = 6), spleen (n = 6), LN (n = 6), liver (n = 6) and lungs (n = 3) of immunized BLT mice. PC-specific IgG, KLH-specific IgM and KLH-specific IgG producing plasma cells were rare in each of the tissues sampled. Statistical analyses were 1 way ANOVA with Bonferroni's multiple comparisons tests. *** indicates a p value less than 0.001.

Finally, we performed ELISPOT analyses using cells isolated from BM, spleen, LN, liver and lungs of immunized BLT mice to quantitate IgM- and IgG-producing plasma cells specific for PC or KLH. The numbers of plasma cells producing IgM specific for PC were similar in all tissues analyzed (p = n.s. for all comparison) (Figure 7-right column). In addition, the numbers of plasma cells producing IgM specific for PC in the BM and liver were significantly greater than the other 3 categories of plasma cells quantitated in each tissue (p<0.001 for all comparisons) which provides evidence of a defect in isotype switching in BLT mouse B cells (Figure 7-bottom row).

## Discussion

Primary antibody deficiency is associated with reduced serum IgG and IgA, often accompanied by reduced serum IgM [12]. These disorders, which include CVID, result from a spectrum of B cell abnormalities caused by various genetic defects which may affect T, B and potentially other cells [3]–[5], [8]–[11]. Here, we examined the suitability of BLT humanized mice to function as an *in vivo* small animal model of primary antibody deficiency. We determined that these patients and BLT mice share remarkable similarities in their humoral immune system development and function.

We began this study with a baseline characterization of humoral immunity in naïve BLT mice. We found that BLT mice exhibited profound hypogammaglobulinemia (Figure 1A) and an absence of memory B cells (Figures 2 and 4). Thus, B cells emigrated from the BM to peripheral tissues, but did not differentiate into unswitched/switched memory B cells or produce IgG. Next, we sought to stepwise verify the stages of normal B cell development in BLT mice. To do this, we performed a comprehensive analysis of B cell development and differentiation in this model. In the BM of BLT mice, we found that B cell ontogeny progressed as it does in normal human BM (Figure 3) [11], [56]–[59]. Together these data show that B cells in BLT mice develop normally in the BM and emigrate to other tissues throughout the body, but B cell maturation is incomplete as the cells do not further differentiate into classical unswitched/switched memory B cells.

This stall in B cell differentiation in BLT mice could be the result of atypical lymphoid architecture in secondary lymphoid tissues that may impede isotype switching (Figure 1C) although recent data suggest that B cell maturation can be completed outside of germinal centers [61]. Alternatively, the stall in B cell differentiation in BLT mice could result from limited antigenic stimulation associated with their specific pathogen-free housing conditions [22], [28]. To address this possibility, we performed repeated PC-KLH immunizations of BLT mice. We observed changes in the B cell populations of the PB and tissues including increased levels of classical unswitched, classical switched and atypical unswitched memory B cells (Figures 5&6). With these increases, we also observed antigen-specific humoral responses that increased over time (Figure 5G) and robust numbers of PC-specific IgM producing plasma cells in tissues (Figure 7). However, we did not observe improvements in lymphoid architecture in the spleen or lymph nodes (Figure 6D), nor did we detect antigen-specific IgG producing plasma cells in tissues (Figure 7). The marginal improvements in B cell function observed in BLT mice following PC-KLH immunization are reminiscent of both immunized CVID patients who may develop IgM, but not IgG, responses to the vaccine antigens [10], [52], [62] and pediatric severe combined immunodeficiency (SCID) patients who receive allogeneic hematopoietic stem cell transplants but do not gain normal B cell function despite multiple and varied antigen stimulations and infections [63]. When taken together these observations indicate that BLT mice exhibit numerous important primary antibody deficiency patient phenotypes and can serve as a robust, small animal model of these disorders in humans.

No single animal model is likely to recapitulate all aspects of primary antibody deficiency in humans. Nevertheless, small animal models could be valuable tools in the efforts to understand, diagnose and treat such primary immunodeficiencies [20]. Herein, we have identified remarkable similarities between the humoral immune systems of primary antibody deficiency patients and those in BLT mice: (i) hypogammaglobulinemia; (ii) normal B cell ontogeny in BM; and (iii) poor antigen-specific IgG response to immunization. Advantages of the BLT model of primary antibody deficiency include the facts that: (i) human B and T cells are interacting *in vivo* in a tractable small animal model; (ii) HLA-restricted T-cells are present to help with B cell isotype class switching [22], [28], [64]–[66]; and (iii) the observed paucity of switched memory B cells is not due a defined monogenic defect but is the expression of a more complex dysregulation. Elucidation of mechanisms responsible for hypogammaglobulinemia in BLT mice may provide insights into the underlying mechanisms responsible for primary antibody deficiency in patients. Because current hypogammaglobulinemia therapy is primarily limited to passive immunoglobulin transfer and treatment of recurrent infections with antimicrobials [15], strategies developed to overcome hypogammaglobulinemia in the BLT model may readily translate into novel clinical interventions that could actively increase antibody production in patients and improve prognoses.
